# Use of therapeutic plasma exchange in children with thrombocytopenia-associated multiple organ failure in the Turkish TAMOF network

**DOI:** 10.1186/cc14209

**Published:** 2015-03-16

**Authors:** E Sevketoglu, D Yildizdas, O Horoz, H Kihtir, T Kendirli, S Bayraktar, J Carcillo

**Affiliations:** 1 Bakirkoy Dr. Sadi Konuk Research and Training Hospital, Istanbul, Turkey; 2 Cukurova University Medical Faculty, Adana, Turkey; 3 Ankara University Medical Faculty, Ankara, Turkey; 4 Haseki Research and Training Hospital, Istanbul, Turkey; 5 University of Pittsburgh School of Medicine, Pittsburgh, PA, USA

## Introduction

Thrombocytopenia-associated multiple organ failure (TAMOF) can lead to high mortality in critically ill children, possibly related to consequences of thrombotic microangiopathy. Plasma exchange therapy may improve thrombotic microangiopathy [1]. The purpose of this observational cohort study is to describe whether there is an association between use of plasma exchange therapy and outcome in the Turkish TAMOF network.

## Methods

We performed a retrospective cohort analysis in patients with TAMOF at three different pediatric ICUs comparing those who received plasma exchange (+) plus standard therapies with those who did not receive plasma exchange (-) and only received standard therapies.

## Results

Among the 42 TAMOF patients enrolled, all had a primary or secondary sepsis diagnosis. Fifteen received plasma exchange therapy (PE(+) group) and 27 received standard medical treatment without plasma exchange (PE(-) group). The mean age was 17.69 months (8.24 to 54.22) in the PE(+) group, and 13.46 months (6.47 to 20.55) in the PE(-) group. Age (*P *= 0.232), gender (*P *= 0.206), thrombocyte count (*P *= 0.09), OFIscore (*P *= 0.111) and Pelod score (*P *= 0.177) on admission were not statistically different between groups. The overall 28-day mortality was higher in the PE(-) group 70.37% compared with 26.67% in the PE(+) group (univariate *P *=0.006; multivariate controlling for Pelod, OFI, PRISM scores and neurological failure *P *= 0.048). Length of stay was increased in the PE(+) group (*P *= 0.004).

## Conclusion

The positive association found between use of plasma exchange therapy and improved survival supports the potential of this therapy in Turkish children with TAMOF. The positive, although less so, associated treatment effect observed after controlling for illness severity provides further rationale for performing a randomized controlled trial in the pediatric Turkish TAMOF network. Sample size calculations call for a 100-patient trial with a *pre hoc *interim analysis after enrollment of 50 TAMOF patients.
